# Supplementary material for: Learning curve and surgical outcome of robotic assisted colorectal surgery with ERAS program
Source: Sci Rep. 2022 Nov 29;12:20566. doi: 10.1038/s41598-022-24665-w (PMC9709162; doi:10.1038/s41598-022-24665-w)
Supplement: Supplementary file 1 — Supplementary Information 1. [file 41598_2022_24665_MOESM1_ESM.zip › LSC Raw data-Ñ[▒K/072/H_ñw╝╨░O▒Kñσ.pdf]

正式摘要(電子簽章：00000188248195)

W52 / W52 ☐英文標題(注意:無英文姓名-請補病人基本檔!) ☐【讀取】憑證

臺中榮民總醫院

出院病歷摘要

轉入號： 轉出號：

[redacted]

(9)入院日期	109/03/17 (2020) 直腸外科 W52 - 092
(10)轉科日期	
(11)出院日期	109/03/24 (2020) 住院天數計 7 日

診斷

- 入院 :D-S Colon ulcer
- 出院 :D-S Colon ulcer s/p laparoscopic anterior resection with anastomosis on 2020/03/18.

主訴

- Bloody stool was noted on 2020/03/07.

病史

- The 44-year-old female denied any systemic disease and had been allergic to pain killer (medication unknown). According to her statement, bloody discharge after defecation 30 minutes was noted on 2020/03/07. Then, she went to our ED for help where abdominal CT was arranged which showed focal segmental wall thickening of descending colon. Besides, colonoscopy was also arranged which revealed tumor at 45cm from anal verge. However, the pathology report disclosed sigmoid colon ulcer but no tumor was seen. There is no other symptoms of fever, chillness, cough, rhinorrhea, chest pain, palpitation, abdominal pain, diarrhea, constipation or dysuria. Under impression of colon Cancer, cT3N0M0, Stage IIA, she was admitted to CRS ward for further evaluation and management.

體檢發現

- S scopy: lesion 45 cm FAV s/p Bx

  CT: lesion in D-S junc.
  - General appearance :
    - chronic ill looking , conscious clear , without cardiopulmonary distress
    - body weight : 53.7 kg , height : 159.3 cm
    - Vital signs自動帶入date 體溫(BT) 脈搏(P) 呼吸(R) 血壓(BP) 身高(BH) 體重(BW) BMI
      20200317 36.7℃ 94次/分 17次/分 134/85mmHg 159.3cm 53.7kg 21.2
    - Vital signs: B.P. 134/85 mmHg , P.R. 94 /min , R.R. 17 /min , B.T. 36.7 ℃
  - HEENT :
    - Eyes: no anemic conjunctiva, no icteric sclera, normal EOM
    - ENT: no otorrhea, no rhinorrhea, no erythematous mucosa, no injected tonsil, no deformity
  - Neck: supple, freely movable, trachea midline,no deformity, no engorged jugular vein , no palpable thyroid or lymph node enlargement
  - Chest:
    - Inspection: Grossly normal, symmetric and fully expansion, no accessory muscle used, no operation scar, no spider angiomata, no gynecomastia
    - Palpation: no palpable mass or nodule, no subcutaneous emphysema, no tenderness
    - Ausculation: clear breathing sound
  - Heart:
    - Percussion: normal heart size
    - Ausculation: regular heart beat, normal S1, S2, no gallop, no murmur
  - Abdomen:
    - Inspection: in
      - flat contour, symetrically, no scar, no stria, no dilated veins, no rashes
    - Ausculation: normoactive bowel sound, no abdominal bruits
    - palpation: soft, no muscle guarding or rigidity, no local tenderness, no rebounding pain, no palpable mass, no palpable pulsation
    - Percussion: no shifting dulllness, no hepatomegaly, no splenomagaly
  -

Peripheral pulsation :	CA	BA	RA	FA	PA	PTA	DPA
Right side :	++	++	++	++	++	++	++/+++
Left side :	++	++	++	++	++	++	++/+++

  - Genito-anus: no tenderness or palpable mass lesion or rectal shelf, normal external genitalia, normal anal tone, brown stool

CRS CONDITION

- Colonoscope report: up to 50cm fav, tumor at 45cm fav, s/p biopsy tattoo
- CEA: 1.52 ng/ml
- Imaging studies(Sono , CT of abdomen and chest Films):
  - 檢驗項目 :CT ABDOMEN-SURVEY 報告日期 :1090311
    主 訴 :
    Result:
    Finding:
    Imaging Report Form for Colorectal Carcinoma (AJCC 8th)

-
  - 1. Imaging Date: 20200309
    Imaging Modality, Imaging by
    CT scan
    Imaging Protocol:
    Post contrast CT of abdomen showed:
    2. Tumor location / Size
    Location:
    Descending
    Size:
    Non-measurable
    3. Tumor invasion
    Tumor cannot be visualized in this imaging study
    4. Regional nodal metastasis
    No
    5. Distant metastasis (In this study)
    No or Equivocal
    6. Other findings:
    * Focal segmental wall thickening of descending colon, axi#45, cor#21-25, colon ca may be compatible.
    > No space occupying lesion over liver, gallbladder, pancreas, spleen and bilateral adrenal gland.
    > bilateral small renal cysts
    > No enlargement of retroperitoneal lymph nodes
    > Bilateral clear basal lung
    > Left adnexal cystic lesion
    > S/p IUD
    > Some small bowel tethered over uterine, endometriosis induced adhension may be considered
    > Bilateral clear basal lung
    * Several sclerotic lesion over right femoral head and acetabulum, suggest follow up.
    Impression:
    1. Colon ca, TxN0M0
    2. Suspected endometriosis induced adhension of small bowel
    3. Others, As above description.

    檢驗項目 :CT, LUNG 報告日期 :1090313
    主 訴 :
    Result:
    Finding:
    CT scan of chest with IV contrast medium administration:
    >Two small ground-glass nodules are located in LUL(#25-0.3cm) and left major fissure(#45-0.2cm). Recommend regular follow up.
    >Small plate atelectasis with focal pleural thickening is located in medial segment of RML(#44-45).
    >An enhancing nodule(#27-1.0cm) is located at 12 o'clock position of visible right breast. Recommend regular follow up at BS OPD.
    >No definite evidence of enlarged lymph nodes can be identified at the mediastinum and bilateral hilum.
    >No definite evidence of significant osteolytic lesion nor osteoblastic lesion can be identified at visible bony structures.
    >Several small cysts(0.3-0.7cm) in visible bilateral kidneys
    >The size of visible bilateral adrenal glands appears within normal limit in this examination.
    Impression:
    >Two small ground-glass nodules in LUL(#25-0.3cm) and left major fissure(#45-0.2cm)
    >An enhancing nodule(#27-1.0cm) at 12 o'clock position of visible right breast

    檢驗項目 :CHEST PA VIEW 報告日期 :1090312
    主 訴 :
    Result:
    Finding:
    1. Normal heart size.
    2. No obvious lesion in bil. lung field.
    3. Intact bony thorax.
    Impression:
    As above description.
-
  - Lung

手術日期及方法

- 手術日期及方法20200318 Laparoscopic Anterior resection and anastomosis - malignant
- 手術結果及發現 :
  - 1. A 2 x 2 cm annular tumor at D-S juction of colon, which invaded to the muscular layer but didn't penetrate through the serosa. Enlarged lymph nodes at mesocolon(-) Borrmann type III, cT3N0M0 2. No other gross tumor nor polyps was found 3. There was no palpable liver metastasis, no visible peritoneal seeding

住院治療經過

- After admission, laparoscopic anterior resection with anastomosis was performed on 2020/03/18. We encouraged her ambulation and removed NG on 3/19. Water was tried on 3/20 and no abdominal pain or fullness was complained. Finally, we let her try soft liquid diet without nausea with vomiting on 3/23. Penrose was removed as little drainage amount. As no specific symptoms or unstable condition, she was discharged on 2020/03/24.

合併症

一般檢查

- 累積報告 :

DATE	TIME	ALB	TP	BIL.T	BIL.D	ALKP	AST	ALT	LDH	CHOL	TG	HDL-C	LDL	UA	NH3	AMY
1090309	1436					52	13									
1090310	1649	4.4		0.8	0.2	56	14	11	157							
1090318	1316	3.6		0.43	0.12	43	14	9								
1090323	0949							5	148							

DATE	TIME	WBC	RBC	HGB	HCT	MCV	PLT	BLST	NEUT	BAND	SEG	LYM	MON	EOS	BAS
1090309	1433	9830		12.6			182		78.0			17.4	3.7	0.7	0.2
1090310	1649	9200	4.13	12.6	36.6	88.6	202		81.2			14.3	3.8	0.4	0.3
1090317	1708	8680	4.43	13.4			252								
1090318	1316	19270		12.5	37.1		177		91.1			7.6	1.0	0.1	0.2
1090323	0901	7470	3.61	11.2	32.6	90.3	244		73.6			17.3	5.1	3.7	0.3

DATE	TIME	NA	K	CL	CA	BUN	CREAT	Mg	P	CRP	ESR	PCT	LIPASE	LACTATE
1090309	1436	141	3.2			9	0.61							
1090310	1649	140	3.5		8.7	10	0.61							
1090317	1708	140	3.9											
1090318	1316	141	4.4		7.7	15	0.57							
1090323	0949		3.7		7.9	9								

DATE	TIME	GLUCOSE	PAB	TRF	ZN	CU	KETONE	CK	CKMB	PT-P	PT-C	APTT-P	APTT-C	TRO-T
1090309	1433									10.9	10.1	28.1		
1090309	1436	79												
1090310	1649	71								10.9	10.1	28.8		

DATE	TIME	PH	BEB	SCVO2	PO2	HCO3	HGB	PCO2	TCO2
1090309	1432	7.334	-2.4	71.4	40.7	23.6		45.4	25.0

- 放射線報告 :檢驗項目 **:CT ABDOMEN-SURVEY** 報告日期 **:1090311**

主　訴：
Result:
Finding:
Imaging Report Form for Colorectal Carcinoma (AJCC 8th)
1. Imaging Date: 20200309
Imaging Modality, Imaging by
CT scan
Imaging Protocol:
Post contrast CT of abdomen showed:
2. Tumor location / Size
Location:
Descending
Size:
Non-measurable
3. Tumor invasion
Tumor cannot be visualized in this imaging study
4. Regional nodal metastasis
No
5. Distant metastasis (In this study)
No or Equivocal
6. Other findings:
* Focal segmental wall thickening of descending colon, axi#45, cor#21-25, colon ca may be compatible.
> No space occupying lesion over liver, gallbladder, pancreas, spleen and bilateral adrenal gland.
> bilateral small renal cysts
> No enlargement of retroperitoneal lymph nodes
> Bilateral clear basal lung
> Left adnexal cystic lesion
> S/p IUD
> Some small bowel tethered over uterine, endometriosis induced adhension may be considered
> Bilateral clear basal lung
* Several sclerotic lesion over right femoral head and acetabulum, suggest follow up.
Impression:
1. Colon ca, TxN0M0
2. Suspected endometriosis induced adhension of small bowel
3. Others, As above description.

檢驗項目 **:CT, LUNG** 報告日期 **:1090313**
主　訴：
Result:
Finding:
CT scan of chest with IV contrast medium administration:
>Two small ground-glass nodules are located in LUL(#25-0.3cm) and left major fissure(#45-0.2cm). Recommend regular follow up.
>Small plate atelectasis with focal pleural thickening is located in medial segment of RML(#44-45).
>An enhancing nodule(#27-1.0cm) is located at 12 o'clock position of visible right breast. Recommend regular follow up at BS OPD.
>No definite evidence of enlarged lymph nodes can be identified at the mediastinum and bilateral hilum.
>No definite evidence of significant osteolytic lesion nor osteoblastic lesion can be identified at visible bony structures.
>Several small cysts(0.3-0.7cm) in visible bilateral kidneys
>The size of visible bilateral adrenal glands appears within normal limit in this examination.
Impression:
>Two small ground-glass nodules in LUL(#25-0.3cm) and left major fissure(#45-0.2cm)
>An enhancing nodule(#27-1.0cm) at 12 o'clock position of visible right breast

檢驗項目 **:CHEST PA VIEW** 報告日期 **:1090312**
主　訴：
Result:
Finding:
1. Normal heart size.
2. No obvious lesion in bil. lung field.
3. Intact bony thorax.
Impression:
As above description.

特殊檢查

病理報告

- 活體組織切片病理報告 手術日期：**109/03/10** 病理號：**S0907408**

  診斷醫師:詹以吉 報告日期:109/03/13
  Pathologic diagnosis:
  Sigmoid colon, endoscopic biopsy --- Ulcer. No tumor seen. See comment.
  Ancillary study for diagnosis:
  Step cut x 3 and P53 (-) and AE1/AE3 (-) IHC stains done. No tumor seen.
  Gross description:
  The specimen consists of 2 pieces of tan soft tissue, up to 0.3x0.2x0.2 cm. All for section.
  Microscopic description:
  Section shows colon mucosa with ulcer. No tumor seen.
  Comment:
  This specimen may not be representative. Further clinicopathological correlation and study are suggested.
  #T-59300_2 #M-38000_2 0 1340 000000

  活體組織切片病理報告 手術日期：**109/03/18** 病理號：**S0908114**

  診斷醫師:陳韻安 報告日期:109/03/25
  Pathologic diagnosis:
  1. Sigmoid colon, laparoscopic anterior resection --- a. Ulcer. b. Unremarkable lymph node (0/17).
  2. IMA root, dissection --- Unremarkable (0/5).
  3. Colon, cut end, distal, resection --- Unremarkable.
  Ancillary study for diagnosis:
  Special stains for PAS (no fungal element seen), Congo red (-) and Masson's trichome (highlight submucosal fibrosis) and IHC stains for AE1/AE3 (highlight epithelial cells) and CMV (-) are done on section C.
  Gross description:
  The specimen consists of 1) a segment of colon, 16.6 cm in length and 4.8 cm and 4 cm in bilateral cut end circumference, respectively, and mesocolic soft tissue. An ulcer, 2.1x1.2 cm, is noted, 2 cm to the smaller cut end. On cross section, the ulcer involves to the submucosa with 48 mm to the radial margin, and serosal retraction is also noted. The both cut ends are free of tumor grossly. 2) IMA root, a piece of tan yellow soft tissue, 4x1.8x1 cm. 3) distal cut end, a piece of tan mucosa-covered soft tissue, 2.6x2.2x1 cm. Representative part for section: A) proximal cut end B) distal cut end C-D, K-L) ulcer (ulcer all for section) E) radial margin F) all of specimen 2 G) specimen 3 H-J) lymph nodes. Blue ink indicated serosa; yellow ink indicated radial margin.
  #T-59300_2 #M-38000_2 0 1105 000000
  #T-C4560_2 #M-09183_2 0 1105 000000

其它

出院時情況

- 1.治癒出院

出院指示

藥名	劑量	劑量單位	用法	頻次	數量	數量單位	現況	天數	單位
Lactam tab 500mg	1	TAB	PO	Q6HPRN	24	TAB	TH	6	天

出院計劃

- 門診日期
  - date on 2020/03/30
- 衛教 :一、飲食攝取：1.少量多餐。2.禁食刺激性食物，如太辣、太腥，大量飲酒及抽太多煙、濃茶和咖啡。3.多攝取高蛋白食物，以加速傷口修復。如：新鮮瘦肉、魚、蛋等。4.並減少吃產氣食物可避免脹氣。如：牛奶、豆類、地瓜、蛋、洋蔥。 二、術後活動：1.三個月內不可從事下列活動：騎馬、騎腳踏車、劇烈的跳舞、久坐及開車，以免骨盆充血。2.手術後 6 ? 8 週內避免提超過5KG之重物，若提物品可使用腹托或束腹帶以免震動所引起之疼痛。3.依個人的體力採漸進增加活動量，可先採輕工作，如無不適，體力許可再增加活動量。 三、傷口護理：1.保持傷口清潔、乾燥，若有不慎弄濕傷口，可用清潔的毛巾或紗布拭乾。2.每日觀察傷口，是否有紅、腫、熱、痛，或不正常的分泌物。 四、居家服藥：1.帶回的藥物，注意藥袋上的服用劑量、時間及注意事項。2.服用軟便劑若解便正常或有腹瀉現象時應停藥或減量。 五、出院後，若有下列情況需立即返院檢查：1.高燒不退。2.腹部疼痛厲害或嚴重腹脹嘔吐。3.傷口流血不止，或是有不正常之分泌物4.有其它問題，請與大腸直腸外科相關人員聯絡 TEL: 04-23592525 轉5161-3 或 6051, 6052

主治醫師 2222H 林俊余 / 住院醫師 9880H 張建林

正式摘要(電子簽章：000000188248195)

W52 / W52

doctorRank:B
